# Neighborhood Density Effects in CSL Sign Recognition: Location, Handshape, and Movement Diverge

**DOI:** 10.3390/bs16101937

**Published:** 2026-10-17

**Authors:** Xiaohong Zhang, Hong Li, Hongwen Cao

**Affiliations:** 1 School of Foreign Languages and Cultures, Chongqing University, Chongqing 400041, China; wendyzhaang@outlook.com (X.Z.); eeehongli@cqu.edu.cn (H.L.); 2 Research Center for Language, Cognition and Language Application, Chongqing University, Chongqing 400041, China

**Keywords:** lexical recognition, sign language, phonological parameters, phonological neighborhood density

## Abstract

Most spoken word recognition models assume that a target word co-activates phonologically similar neighbors that compete for recognition, a process reflected in inhibitory neighborhood density effects. Sign languages provide a critical test for the generality of these mechanisms with simultaneously articulated phonological parameters like location, handshape, and movement, as well as prevalent iconic form-meaning mappings. The present study employed a lexical decision task in Chinese Sign Language to investigate how density defined over each parameter affects recognition and whether subjective lexical frequency modulates these effects. Results showed that location density inhibited reaction times for both signs and non-signs, consistent with competition among lexical representations activated through shared sublexical location features. Handshape density facilitated accuracy selectively for lower-frequency signs, probably reflecting a late decisional strategic bias driven by the familiarity of typical handshape values under weak competition. Movement density showed no significant effects, while subjective lexical frequency robustly facilitated reaction times and accuracy, validating task sensitivity. The results suggest that parameters are used differently during lexical sign recognition, likely due to different perceptual timing and resting activation levels. They are also consistent with spoken word recognition principles of neighbor competition and frequency modulation, implying some shared lexical recognition mechanisms across modalities.

## 1. Introduction

Lexical recognition is the process of mapping incoming sensory information (acoustic or visual signals) onto lexical representations stored in long-term memory and thus activating phonological, semantic, and syntactic properties of these representations (Dahan & Magnuson, 2006; Magnuson et al., 2012). It is a core component of language comprehension and forms the basis for fluent daily communication. This process seems to unfold rapidly and effortlessly within a few hundred milliseconds, but involves complex decoding processes. Uncovering the mechanisms underlying lexical recognition can not only illuminate the fundamental cognitive architecture of human language processing but also provide practical implications for language acquisition, language disorder assessment, and educational interventions (Fernald & Frank, 2012; Magnuson et al., 2007; Pisoni, 2018).

Over the past decades, psycholinguists have employed a wide range of paradigms and techniques to deepen our understanding of this process. A set of influential theoretical and computational models have been proposed for lexical recognition in spoken languages (e.g., the cohort model, Gaskell & Marslen-Wilson, 1997, 2002; the neighborhood activation model, Luce & Pisoni, 1998; TRACE, McClelland & Elman, 1986; Shortlist, Norris, 1994; Norris & McQueen, 2008; EARSHOT, Magnuson et al., 2020; for reviews, see Dahan & Magnuson, 2006; Weber & Scharenborg, 2012). Most of the models assume that the acoustic speech input is initially converted into phonetic attributes which are then organized into sublexical units like phonemes and syllables. The sublexical units subsequently map onto lexical entries stored in the mental lexicon, leading to word recognition. During this process, multiple lexical entries compatible with the acoustic signal would be activated and compete with the target word for recognition. This has been evidenced in part by the impact of phonological neighborhood density (PND), a measure of the number of phonologically similar words, on spoken word recognition performance. A wide range of studies have shown that words in a dense neighborhood elicited slower reaction times (RTs) and/or lower accuracy (Dufour & Frauenfelder, 2010; Dufour & Peereman, 2003; Goh et al., 2009, 2016; Luce & Large, 2001; Luce & Pisoni, 1998; Magnuson et al., 2007; Vitevitch, 2002) or larger event-related potentials (ERPs) amplitude (Dufour et al., 2013; Hunter, 2013; Strauß et al., 2022; Winsler et al., 2018) compared to words in a sparse neighborhood. These inhibitory effects are generally attributed to increased interference or competition from phonologically similar words in dense neighborhoods.

Some other studies, however, reported reversed facilitatory effects of PND on RTs (Ferrand et al., 2018; Vitevitch & Rodríguez, 2005) or early ERP components (Dufour et al., 2013). These effects are assumed to reflect the impact of phonotactic probability, a conceptually related variable which measures how frequently segments and sequences of segments occur in the lexicon (Vitevitch et al., 1999; Vitevitch & Luce, 2004). PND is normally positively correlated with phonotactic probability, such that words with many neighbors tend to be composed of more frequent segments and sequences. In contrast to the typical inhibitory effect of neighborhood density, high phonotactic probability typically facilitates processing, especially for non-words and low-frequency words (Luce & Large, 2001; Vitevitch, 2003; Vitevitch et al., 1999; Vitevitch & Rodríguez, 2005). This contrast has been hypothesized to result from processing at different levels: dense phonological neighborhood is assumed to engage lexical-level competition (inhibition), whereas high phonotactic probability facilitates sublexical-level mapping (facilitation), especially when lexical competition is weak (Strauß et al., 2022; Vitevitch & Luce, 1999, 2016).

The effect of neighborhood density or phonotactic probability can serve as a tool for probing the psychological reality of sublexical units. PND is normally measured on the basis of shared sublexical units across words. An effect of density can only arise if the relevant units are represented in the mental lexicon and activated during processing, yielding activation of lexical candidates compatible with these units. By observing how density defined over a particular sublexical unit affects lexical recognition, we can infer whether that unit is activated and participates in competition during lexical access.

**Figure 1:**
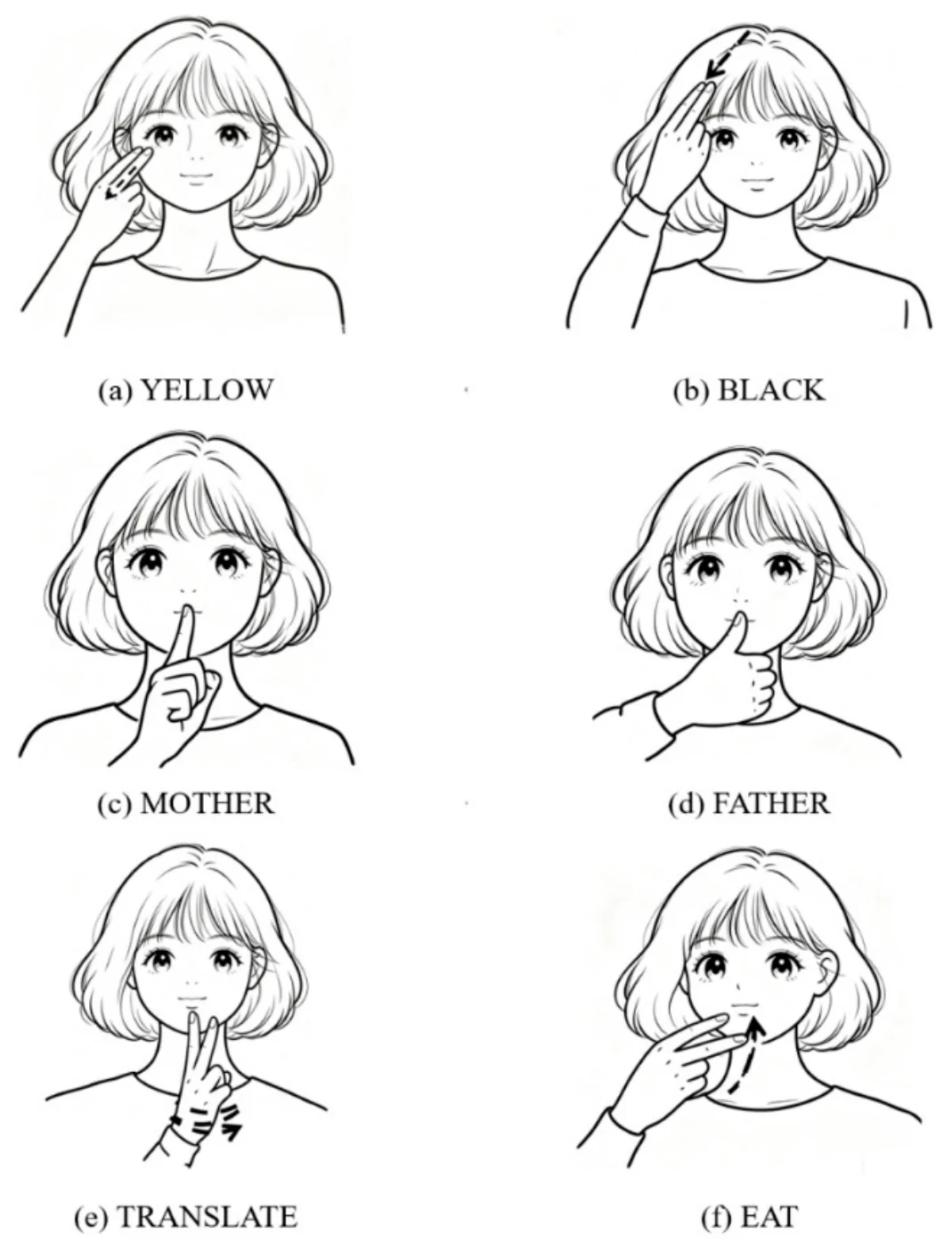
Three minimal pairs in CSL. (**a**) YELLOW and (**b**) BLACK differ by virtue of location; (**c**) MOTHER and (**d**) FATHER differ by virtue of handshape; (**e**) TRANSLATE and (**f**) EAT differ by virtue of movement.

As with spoken languages, sign languages are natural human languages with systematic phonological structures and contain lexical forms that may be similar to each other based on shared phonological units, typically referred to as phonological parameters in sign language research. Location (the position of the hands relative to the body), handshape (the general shape of the hands), and movement (changes in hand position, wrist joints, or fingers) are the major phonological parameters comprising lexical signs (Fenlon et al., 2018; Johnson & Liddell, 2010; Sandler, 2017; van der Kooij & Crasborn, 2008) and are often used to operationalize phonological similarity or neighborhood in sign languages (Carreiras et al., 2008; Caselli et al., 2017; Gutiérrez et al., 2012a; Meade et al., 2022). The parameters combine in rule-governed ways to form signs with different meanings and are contrastive in a way similar to spoken language phonemes, so that changing one parameter could result in minimal pairs of signs. Figure 1 provides graphic representations of three minimal pairs in Chinese Sign Language (CSL), where each pair differs by virtue of only one parameter. Unlike the mostly sequential organization of spoken phonemes, the parameters unfold to a large extent simultaneously during sign articulation. In addition, signs manifest a larger degree of iconicity than spoken words; that is, many sign forms visually resemble or bear a motivated relation to their meaning (Dingemanse et al., 2015; Hou, 2018; Ortega et al., 2025; Wang, 2024). These unique properties raise the question of whether the parameters and related neighbors are activated in sign recognition in a similar vein to spoken word recognition.

Initial evidence from sign languages shows that PND also modulates lexical access and recognition of signs. Caselli et al. (2021) reported an inhibitory effect of neighborhood density, defined as the number of signs sharing all three major parameters, in a lexical decision task, where participants were presented with a bunch of stimulus videos and required to decide as quickly and accurately as possible whether the stimulus was a real sign in American Sign Language or not. Signs with many neighbors were recognized more slowly and less correctly than signs with few neighbors, but only for signs with lower subjective frequency ratings. The results are broadly consistent with inhibitory effects of neighborhood density in spoken word recognition.

However, the neighborhood density effects diverge when neighbors are defined in terms of overlap in a single parameter. Signs sharing location values are referred to as location neighbors, signs sharing handshape values as handshape neighbors, and signs sharing movement values as movement neighbors. Carreiras et al. (2008) found that signs with many location neighbors in Spanish Sign Language were responded to more slowly than those with few location neighbors, while signs with many handshape neighbors were recognized faster than those with few handshape neighbors (the latter limited to non-native signers). Again, the effects were restricted to less frequent signs. Facilitatory effects of handshape neighbors were also reported in Taiwan Sign Language lexical recognition in a same-different matching task, in which deaf signer participants needed to judge whether two consecutively presented signs were the same or not (Ann et al., 2011).

Caselli and Cohen-Goldberg (2014) simulated the reversed effects of location and handshape neighborhoods in a computational model by incorporating different temporal dynamics and resting activation levels of the parameters. Location was argued to be perceived earlier than handshape and movement (Emmorey & Corina, 1990) and have a higher resting activation level due to higher visual salience (Orfanidou et al., 2009), earlier acquisition (Bonvillian & Siedlecki, 2000; Conlin et al., 2000; Karnopp, 2002; Marentette & Mayberry, 2000; Morgan et al., 2007; Ortega & Morgan, 2015; Siedlecki & Bonvillian, 1993), and higher sublexical frequency. Sublexical frequency matters because, when neighbors are defined in terms of overlap in one sublexical parameter, neighborhood density is almost identical to the frequency of that parameter in the lexicon, and location frequency on average was found to be several times larger than the average handshape frequency (Caselli et al., 2017; Gutierrez-Sigut et al., 2016). Consequently, location neighbors were strong competitors with longer activation windows and higher resting levels, producing inhibition. Handshape neighbors, in contrast, were weak competitors with short activation and lower resting activation levels, eliciting facilitation. The two hypotheses successfully simulated Carreiras et al.’s (2008) findings.

However, other studies in sign recognition have reported mixed results on effect directions of the parameters, especially those using the priming paradigm. Effects of location processing are relatively consistent, with inhibition on recognizing sign targets when they were preceded by primes with shared location (Corina & Hildebrandt, 2004; Gutiérrez et al., 2012a, 2012b) and interference on overall looking time to the target from pictures whose corresponding signs shared location with the target (Villameriel et al., 2019, 2022). Findings on handshape processing are more complex. Some studies reported facilitatory priming effects of handshape overlap, as is with handshape neighborhood effects (Meade et al., 2021, 2022), whereas other studies showed either reversed inhibitory (Gutiérrez et al., 2012a) or no significant (Dye & Shih, 2006) priming effects of handshape overlap. Movement effects have received the least attention. To the best of our knowledge, only one study has reported a significant independent effect of movement processing, where movement overlap between prime and target elicited facilitation on target recognition for deaf non-native signers of British Sign Language (Dye & Shih, 2006), though studies exist showing combining effects of movement and other parameters (R. L. Thompson et al., 2013; Wienholz et al., 2021). No study to date has investigated the influence of movement neighborhood density on sign recognition. These mixed or limited results warrant further explorations on how each parameter contributes to lexical sign recognition.

The present study builds upon and extends Carreiras et al.’s (2008) study to systematically investigate the effects of location, handshape, and movement neighborhood density on lexical recognition in CSL. A neighborhood density approach can help determine whether phonological parameters and relevant similar lexical representations are spontaneously activated during sign recognition while avoiding potential confounds of visual or iconic overlap between specific prime-target pairs that can arise in priming studies (Caselli et al., 2021). Lexical frequency and its interactions with parameter neighborhoods were also investigated. The lexical frequency effect has been well-established in lexical recognition research, with higher-frequency words responded to with shorter RTs and/or more accuracy than those with lower frequency (Brysbaert et al., 2018; Ernestus & Cutler, 2015; Ferrand et al., 2018; Hauk & Pulvermüller, 2004), which can serve as a test case for the reliability of the current task. Interactions between lexical frequency and parameter neighborhood density were investigated, as previous studies in both spoken word and sign recognition showed neighborhood density effects were stronger for less frequent words or signs (Carreiras et al., 2008; Caselli et al., 2021; Goh et al., 2009; Prabhakaran et al., 2006; Strauß et al., 2022).

The present study contributes to the literature in the following ways. For one thing, it extends investigations of location and handshape processing into a novel sign language (CSL) and, importantly, incorporates movement neighborhood density for the first time. This provides a new empirical test of Caselli and Cohen-Goldberg’s model across all three major phonological parameters. For another, data were analyzed here on both real signs and non-signs to examine whether density effects arise solely from lexical-level competition among existing lexical entries, or whether they also reflect sublexical processing of the parameters. As is suggested in prior spoken language studies (e.g., Bien et al., 2015; Dumay et al., 2001; Hunter, 2013; Norris et al., 2002; Slowiaczek et al., 2000), different effects of phonological similarity between signs and non-signs suggest processing at the lexical level, as non-signs do not have lexical representations. Otherwise, if signs and non-signs demonstrate similar effects, they likely originate from sublexical processing normally resulting in facilitatory effects. In addition, the study departs from the common practice of dichotomizing density into high versus low categories in previous studies. Instead, we treat parameter density as a continuous predictor within linear mixed-effects (LME) models fitted to trial-level data. This avoids possible issues of experimenter bias in setting boundaries of an inherently continuous variable, maximizes statistical power, simultaneously accounts for by-subject and by-item random variance, and can model multiple variables while controlling collinearity (Brown, 2021; Emmorey et al., 2020; Ferrand et al., 2018; Matuschek et al., 2017).

Through these methods, the present study attempts to answer the following questions:

(1) Do location, handshape, and movement neighborhood density respectively affect RTs and accuracy in CSL lexical decision? If so, does the direction of effects differ across parameters?
(2) Does lexical frequency modulate these parameter-specific density effects, that is, do density effects differ between high- and low-frequency signs?
(3) Are these density effects comparable across real signs and non-signs, that is, do they reflect lexical-level competition or do they also affect sublexical processing?

Based on Caselli and Cohen-Goldberg’s (2014) model, if location, due to its early perception and high resting activation level, comprises strong competitors, its density may produce inhibitory effects. If handshape constitutes weak competitors, its density may produce facilitatory effects, especially under weak lexical competition, e.g., for low-frequency signs or non-signs, or may show no significant effect. Though the model does not provide separate predictions for movement, we predict that movement may comprise weak competitors and produce facilitatory effects like handshape neighbors given its late perception (Emmorey & Corina, 1990). Lexical frequency may moderate these effects, such that weak-competitor effects are more pronounced for low-frequency items. The findings may not only clarify how sublexical phonological units are used during lexical access in the visual-gestural modality, but also inform the generalizability of existing spoken language models across sign languages.

## 2. Methods

### 2.1. Participants

Twenty–three deaf CSL signers (13 males and 10 females) aged 17–28 years (M = 22.26, SD = 2.36) participated in the study. Five of them were congenitally deaf and the rest were diagnosed with severe or profound hearing loss at the age of 1–7. Three of them were born to deaf parents but did not begin to acquire CSL from birth (age of CSL exposure at 4, 5, and 10 years). The remaining 20 participants were born into hearing households with a wide range of ages at first CSL acquisition. In total, the participants were exposed to CSL from the age of 4 to 14 (M = 8.04, SD = 2.93).

All participants were recruited from Chongqing, China. They were either attending or had graduated from the Special Education College, Chongqing Normal University, or the Deaf School of Chongqing at the time of testing. They tended to use CSL, signed Chinese, and written Chinese in class, but preferred to use CSL for communication with friends and families outside class. By the time of testing, they reported that they had been using CSL as the primary and preferred language for at least 9 years (range = 9–24, M = 15.22). Each participant completed a questionnaire on their CSL and Chinese use, parents’ hearing status, language preference, etc. They were all right-handed with normal or corrected visual acuity. Each signed a written informed consent before the experiment and received monetary compensation after the experiment. For the 17-year-old participant, written informed consent was also obtained from a parent.

### 2.2. Materials

Materials for the experiment were selected from a CSL lexical database created specifically for psycholinguistic studies in CSL (Zhang et al., in preparation), which contained over 600 CSL signs and 800 non-signs. Signs in the lexical database were selected from the latest and largest standardized CSL dictionary (China Association of the Deaf and Hard of Hearing & National Research Center for Sign Language and Braille, 2019) and restricted to citation forms. Compound signs and bimanual signs with different information on the two hands were excluded. Fingerspelled signs and classifier constructions were included only if they were lexicalized and commonly used by CSL deaf signers. Location and handshape of each sign were annotated by the first author following the inventory described by Yi (2008), who offered the first in-depth analysis of phonological parameters in CSL and has provided detailed descriptions of categorizing and coding the parameters. Annotations of movement were instead based on a lexical database of American Sign Language (Sehyr et al., 2021), as Yi’s (2008) movement annotations were different from the categorization of movement in most phonological models and psycholinguistic studies. The full set of annotations was subsequently verified by a proficient hearing signer with extensive long-term CSL experience; discrepancies were resolved by consensus.

Non-signs in the database were created by substituting one major parameter of a real sign while keeping the other parameters unchanged. The parameter to be changed was predetermined for each sign to balance the number of substitutions across parameters. Some signs yielded more than one non-sign. To match real-sign criteria, non-signs appearing to be compounds (involving changes in major location and path movement; Brentari, 1998) or with different handshapes in two hands, were excluded. The signing model and the proficient hearing CSL signer evaluated the non-signs and those noted as likely to be mistaken for real signs were excluded.

A deaf male native signer produced all materials at a natural speed. He wore a black shirt and performed the signs in front of a gray background to enhance contrast. Non-manual features such as mouth movements and facial expressions were only included when they formed an essential part of a sign. The video recordings were edited using Microsoft Photos software (version 2020.20120.4004.0) on Windows 10. Sign onset was defined as the first frame where the primary handshape and location were clearly established, and offset as the last frame where the handshape remained distinct before the hands moved to a resting position (Gutiérrez et al., 2012a; Gutierrez-Sigut et al., 2016). The onset of the video clips was cut at 6 frames before the sign onset in case the initial phonemic information was blurred due to fast transitioning movement from the resting position to sign onset, but the offset of the videos coincided with that of the signs. Each video clip was 960 pixels wide by 540 pixels high, with a frame rate of 30 frames per second.

The full database and its construction will be described in a separate publication, but item-level metadata for all stimuli used in the present study are provided in the Supplementary Materials (Data S1). For the present study, 351 real signs as well as 351 non-signs were selected. Signs selected were all monomorphemic with a single major location, a constant selection of fingers, and a single path movement (Brentari, 1998; Fenlon et al., 2018; van der Kooij & Crasborn, 2008). This can ensure that any effects found for parameter activation can be attributed to the given parameter. With parameter changes, e.g., two major location values in a compound sign, it is hard to decide whether the effects were caused by the first or second location. The non-signs also appeared “monomorphemic” in form, without changes in major location or path movement and with consistent handshape values in two hands. Their location, handshape, and movement were coded so that parameter neighborhood density for each non-sign can be calculated based on the lexical database. Real signs and non-signs differed significantly in duration (real: M = 852 ms; non-sign: M = 1089 ms; *t*(683.38) = −8.81, *p* < 0.001, Cohen’s *d* = −0.66). Duration was therefore included as a continuous covariate in all analyses.

Characteristics of the stimuli used in this study are summarized in Table 1. They were drawn from the CSL lexical database. Parameter neighborhood density was calculated as the number of signs in the database sharing a specific parameter value, e.g., the number of signs articulated in the neutral space, the number of signs with a 5 handshape, or the number of signs in a straight path movement, in line with operationalizations in Carreiras et al.’s (2008) study. Three types of movement neighborhood density were used, considering controversies in movement representations in sign language phonology (Brentari, 1998; Sandler, 1986; van der Kooij, 2002) and limited investigations into its role in online processing. One measured movement density as the number of signs sharing both path movement (movement of the whole hand from one point to another) and hand internal movement (movement in the wrist or fingers, or a rotation of the fingers or lower arm), consistent with the treatment of movement as a unitary phonological parameter in the Prosodic Model of sign language phonology (Brentari, 1998); another used neighborhood density comprising path and hand internal movement as two separate predictions, and the last one considered path movement only, following the studies available on movement processing (e.g., Dye & Shih, 2006).

Objective measures of lexical frequency based on large-scale corpora are not available now in CSL, so subjective ratings of lexical frequency by deaf signers were used instead, as practiced in many other psycholinguistic studies in sign languages (e.g., Carreiras et al., 2008; Novogrodsky & Meir, 2020; Sehyr et al., 2021; Trettenbrein et al., 2021). Such subjective ratings have been found to be highly correlated with corpus–based frequency estimates in both spoken/written words (Ferrand et al., 2008; G. L. Thompson & Desrochers, 2009) and signs (Fenlon et al., 2014) and be able to capture frequency effects in spoken/written languages (see Ernestus & Cutler, 2015 for a megastudy on effects of different measures of frequency). In addition, the ratings are relatively stable across proficient deaf signers irrespective of their age of acquisition (AoA), chronological age, or years of acquisition (Mayberry et al., 2014).

Twenty-nine deaf CSL signers (15 male; mean age = 22.38 years, SD = 2.50 years) rated each sign on a 7-point scale based on how frequently they used it in daily communications (1 = very rarely, 7 = very frequently). None of these signers were invited to participate in the present experiment. Inter-rater reliability was assessed using intraclass correlation coefficients (ICC, two-way random effects, absolute agreement, average measures) with the psych package (version 2.6.9; Revelle, 2026) in R (version 4.4.3; R Core Team, 2025). The ICC(2, k) was 0.986, with a 95% confidence interval (CI) from 0.983 to 0.989, F(350, 9800) = 92.6, *p* < 0.001, indicating excellent agreement among raters. The mean rating across the raters was used as the subjective frequency value for each sign. Objective frequency measures for the spoken equivalents of the signs were also taken from SUBTLEX–CH, a database that provides word and character frequencies derived from film and television subtitles (Cai & Brysbaert, 2010). There is a significant correlation between the subjective ratings and the objective counts (r = 0.353, *p* < 0.001). This modest correlation may be partly attributable to the restricted range of frequency values in the lexical database, which skewed toward higher frequency, but it suggests that participants’ ratings could, at least to some degree, serve as a reasonable proxy for frequency estimates.

Iconicity and length of the signs were also analyzed as covariates to control their potential influence on response RTs and accuracy. Iconicity, i.e., a motivated relationship between form and meaning, is uniquely prevalent in sign languages and has been found to be associated to some extent with parameter density in the lexicon (Caselli et al., 2017; Sehyr et al., 2021). It thus needs to be controlled when examining the impact of phonological parameters. A total of 31 hearing sign-naïve Chinese speakers (9 male; mean age = 21.81 years, SD = 2.63 years) rated how the sign form looked like what it meant from 1 to 7 (1 = not iconic at all, 7 = very iconic). Sign length was calculated as the duration (ms) from sign onset to offset.

### 2.3. Procedure

**Figure 2:**
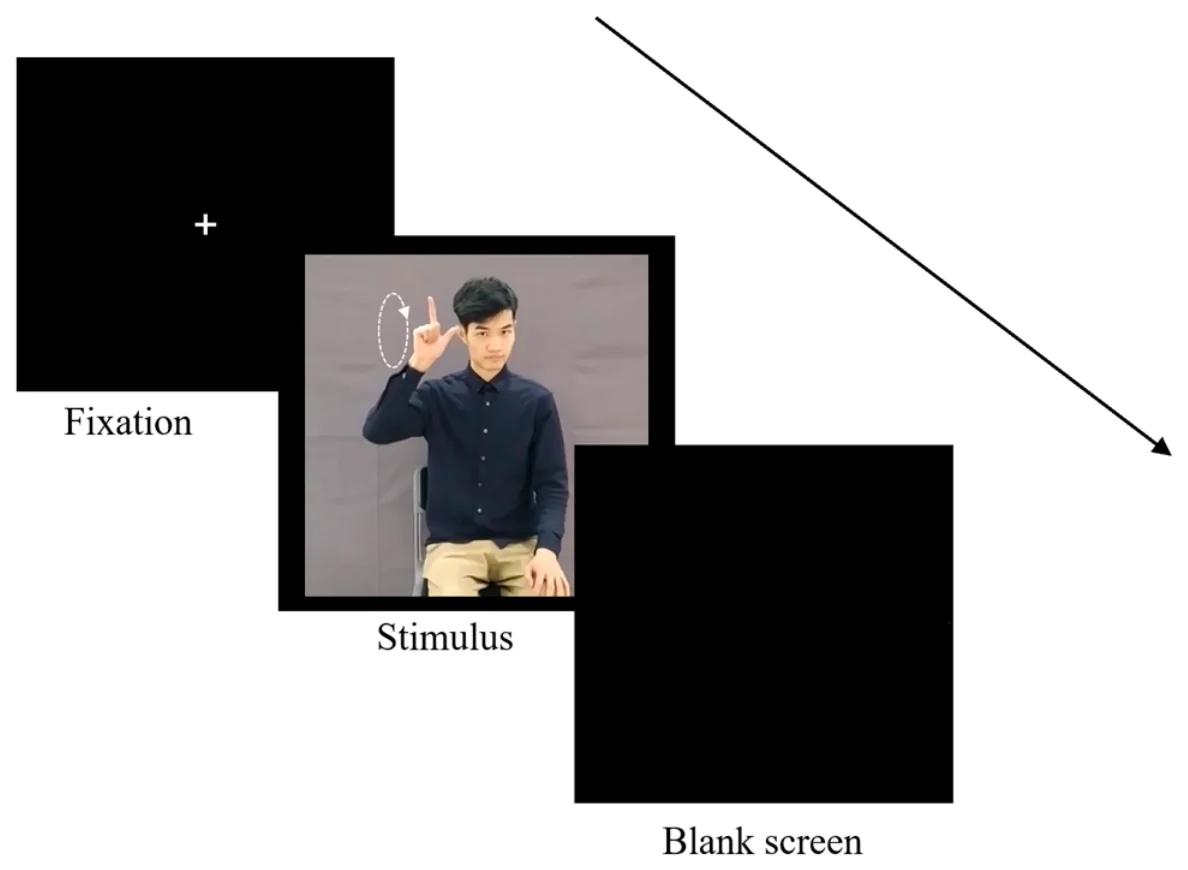
Illustration of the presentation sequence of a trial. The stimulus was displayed here statically as a picture only for illustration; all stimuli in the experiment were presented dynamically in the form of videos.

Participants performed a lexical decision task while sitting comfortably in front of a computer in a quiet room. The experimental software E–Prime 3.0 (Psychological Software Tools) was used for stimulus presentation and data collection. On each trial of this experiment, a white fixation mark (+) appeared first at the center of a black computer screen for 500–800 ms (see Figure 2 for illustration). Then a stimulus video (the target) was presented, after the end of which a blank screen followed immediately. An interval was fixed at 3000 ms between stimulus onset and the blank screen offset, but the duration of both the video and blank screen varied due to differing sign length. Participants were instructed to decide as fast and accurately as possible whether the stimulus was a real CSL sign or not, by pressing the C key on the keyboard if it was a real sign and pressing the M key if it was not. The target stimulus disappeared when the participant responded. RT was measured as the period from stimulus onset to when a response was made. Instructions and explanations of a non-sign were given in both written Chinese and CSL (a hearing proficient CSL signer helped with the explanations), together with pictorial illustrations of a trial. Each participant practiced with twenty trials (10 real signs and 10 non-signs) before the experiment, during which time accuracy feedback was given. No feedback was provided in the formal experiment. The stimuli were presented in a pseudo-randomized order so that each participant received a different order of trials. Participants could take a break as long as they wanted after approximately every 175 trials. The whole experiment took about 20 min.

### 2.4. Statistical Analysis

Data were first trimmed by removing trials that received no response or had slower responses than three standard deviations above the mean RT. Trials with incorrect responses were also removed from the reaction time analyses. The remaining data (see Supplementary Materials, Data S2) were analyzed with LME models using the lme4 package (version 1.1.37; Bates et al., 2015) and the lmerTest package (version 3.1.3; Kuznetsova et al., 2017) in the software R (version 4.4.3; R Core Team, 2025). LME models have been increasingly used in psycholinguistic data analyses, for advantages such as simultaneous modeling of random effects across participants and items, a better way of handling missing data and unbalanced designs, incorporating inherently continuous variables while avoiding arbitrary categorizations, and modeling multiple variables while controlling collinearity (Brown, 2021; Heise et al., 2022; Matuschek et al., 2017; Tibon & Levy, 2015). The last two advantages are particularly important for the current design because multiple continuous variables were investigated.

Data on signs and non-signs were analyzed separately, considering the complexity of the model structure in the current design. For both signs and non-signs, two sets of LME models were structured to find out the effects of parameter density and subjective lexical frequency. One used RTs from each participant and item as the dependent variable, and the other used accuracy per participant and item (coded as Correct = 1, Error = 0). The two sets had almost the same structure except that different regression types were used (linear for RT vs. logistic for accuracy). R scripts for main analyses are listed in the Supplementary Materials (Script S1).

For signs, each model included location, handshape, and movement density as well as subjective lexical frequency as main predictors. Due to the lack of consistency in the linguistic treatment of movement (Brentari, 1998; Sandler, 1986; van der Kooij, 2002), different types of movement density were used in separate LME model analyses. The first analysis combined path and hand internal movement into a single movement category, in line with the Prosodic Model (Brentari, 1998). The second analysis added path and hand internal movement density as two separate predictors. The third analysis considered path movement only, as was practiced in prior studies on movement processing (e.g., Dye & Shih, 2006). Model comparison was based on the differences in Akaike Information Criterion (AIC), which is an information-theoretic measure for evaluating the relative adequacy of statistical models for a given dataset by considering both goodness of fit of the data and model complexity, with lower values indicating better-approximating models (Burnham & Anderson, 2004; Ernestus & Cutler, 2015). By convention, differences in AIC (ΔAIC) ≤ 2 indicate that competing models are essentially indistinguishable in fit, in which case the more parsimonious model is preferred; 4 ≤ ΔAIC ≤ 7 indicates considerably less support for the candidate model as the best approximating model; ΔAIC > 10 indicates essentially no support (Burnham & Anderson, 2004; Symonds & Moussalli, 2011). To ensure comparability of information criteria, the LME models on RTs were fitted using maximum likelihood (REML = FALSE), as they differed in fixed effects (different types of movement density were used) and AIC values obtained under REML are not comparable across different fixed-effect structures. For the accuracy models, generalized linear mixed models were fitted using the glmer function with a binomial link, which estimates parameters via maximum likelihood; AIC values are therefore directly comparable across models with different fixed effects.

Frequency was added to the models because it has robust effects in word recognition and any psycholinguistic studies in language processing that do not manipulate or control it could be doubted. It is also used as a benchmark for lexical processing, as it is a well-established predictor of spoken word recognition and has been assumed to have its locus at the level of lexical processing (see Brysbaert et al., 2018). Two-way interactions between each parameter density and subjective lexical frequency were also added in the models, considering previous research showing interactive effects of neighborhood density and lexical frequency in both spoken word (Goh et al., 2009; Prabhakaran et al., 2006; Strauß et al., 2022) and sign recognition (Carreiras et al., 2008; Caselli et al., 2021). For non-signs, each model only included location, handshape, and movement density as main predictors.

Models on real signs additionally included sign iconicity, duration, participants’ AoA, and trial number as covariates to control their potential influence. Models on non-signs included only duration, participants’ AoA, and trial number as covariates, as iconicity is not available for them. Trial number was added because participants may speed up over the course of the experiment as they get used to the task, or slow down because of fatigue (Caselli et al., 2021). Standardized z-scores were used in the models for all phonological and lexical variables to reduce collinearity (Jevtović et al., 2020). VIF (variance inflation factor) of each variable was also measured to assess multicollinearity among them. The values were all below 5, the typically acceptable threshold, indicating that each predictor’s effects are independent of the others.

All models contained random effects to capture variance across individual participants and items. They were first structured to be maximal (Barr et al., 2013), including random intercepts for participants (variability in RTs or accuracy across participants) and items (variability in RTs or accuracy across items), random slopes for all within-participant variables (variability in response to different lexical variables across participants) as well as covariance or correlations between random intercepts and slopes. If the full model failed to converge, the random effect structure was simplified in a backward way by first removing the correlations between random intercepts and slopes and then the random slopes explaining the least variance sequentially until the model fully converged (Jevtović et al., 2020).

To assess model fit, both the marginal R^2^, representing variance explained by fixed effects alone, and the conditional R^2^, representing variance explained by the full model (fixed + random effects) were calculated using the MuMIn package (version 1.48.19; Bartoń, 2026). Effects of the parameter density and subjective lexical frequency were confirmed using likelihood ratio tests, where the full model was compared with a reduced or null model that lacked the effect of interest but was otherwise the same as the full model (Brown, 2021). For example, to test the effect of location neighborhood density, the full model was compared with a model without the effect of location neighborhood density. If the test resulted in a significant difference (*p* < 0.05), it indicates that the full model containing the effect of interest offers a better fit for the data than the reduced model, hence a significant effect of the variable. The likelihood ratio tests were conducted in the mixed() function from the afex package (version 1.4.1; Singmann et al., 2024). Unstandardized regression coefficients (B), standard errors (SE), 95% CIs, t-values, and *p*-values were summarized using the sjPlot package (version 2.8.17; Lüdecke, 2024) for the parameters in question, and standardized coefficients (β_std) were computed using the effectsize package (version 1.0.3; Ben-Shachar et al., 2020).

To examine whether the key effects observed were driven by any single participant, leave-one-subject-out sensitivity analyses were conducted. For each iteration, one participant was excluded, the main model was refitted, and the coefficient and significance of the critical effects were recorded. This analysis was conducted if significant effects were observed in a given LME model.

## 3. Results

Different sets of models were structured for RTs and accuracy data and for real signs and non-signs, so results will be presented here according to these different sets of models.

### 3.1. Results on Reaction Times

#### 3.1.1. Signs

Results on RTs of real signs centered here on the model using movement neighborhood density combining path and hand internal movement, which showed a lower AIC than both the model using path and hand internal movement as separate predictors (ΔAIC = 30.38) and the model considering path movement only (ΔAIC = 28.33). Following conventional criteria (Burnham & Anderson, 2004; Symonds & Moussalli, 2011), ΔAIC > 10 indicates essentially no support for the competing models relative to the best-fitting model. This pattern not only conforms to the theoretical rationale for adopting the combined measure, i.e., the treatment of movement as a unitary phonological parameter in the Prosodic Model on sign language phonology (Brentari, 1998), but maintains consistency with the parameter-level operationalization used for location and handshape, allowing the three parameters to be compared on equal footing.

The LME model using the combined movement measure yielded a marginal R^2^ of 0.106 and a conditional R^2^ of 0.588. The relatively modest marginal R^2^ is typical for behavioral RT data, reflecting the inherent noisy nature of human responses. Critically, the substantial increase in conditional R^2^ underscores the effectiveness of our random-effects structure in accounting for inter-participant and inter-item variability, enhancing the reliability of the fixed-effect estimates. Other result statistics are summarized in Table 2.

For the phonological parameters, a significant main effect was observed for location neighborhood density. RTs for CSL signs increased with larger location neighborhood density. No significant main effects were found for neighborhood density comprising handshape or movement. Interactions with subjective lexical frequency were nonsignificant for any of the three parameter densities. There was a significant main effect of subjective lexical frequency on RTs, with more frequent signs responded to faster than less frequent signs. The leave-one-subject-out analysis confirmed that the significant effects were robust to individual participants. Both the location density and subjective lexical frequency effects remained significant in all 23 iterations.

For the covariates, there was a significant effect of sign duration, with longer signs responded to more slowly than shorter signs, in line with previous findings (Caselli et al., 2021; Ernestus & Cutler, 2015; Ferrand et al., 2018; Goh et al., 2016). The covariate of trial number also had a significant impact. Participants responded to trials presented later in time more quickly than trials presented earlier, suggesting that they may gradually get more familiar with the task procedure over the course. There were no significant effects of the other two covariates, i.e., sign iconicity and participants’ AoA.

#### 3.1.2. Non-Signs

The three movement operationalizations yielded highly similar AIC values in the LME models on non-sign RTs (model with combined movement: −11,301.21; model with path and hand internal movement as separate predictors: −11,299.04; model with path movement only: −11,301.03; maximum ΔAIC = 2.17). All ΔAIC values were at or below the conventional threshold of 2, indicating that the models are essentially indistinguishable in fit. All three models were checked for results, and they all showed significant effects of location density, stimulus duration, and trial number, while showing no significant effects of other predictors, suggesting that the results are robust to how movement is defined. Results of the model with combined movement were summarized and presented in Table 3 for illustration and for better comparison with results on real sign RTs.

The model yielded a marginal R^2^ of 0.079, which falls within the typical acceptable range for behavioral RT experiments, and a conditional R^2^ of 0.592, indicating that the random effects effectively partitioned participant- and item-level systematic variability. Non-signs with a larger location density were rejected more slowly than those with a smaller location density. The leave-one-subject-out analysis showed that this effect remained significant in all 23 iterations. Effects of the two covariates, i.e., stimulus duration and trial number, were consistent with results on real signs. Non-signs with longer durations had longer RTs than those with shorter durations, and non-signs presented later in time were responded to more quickly than those presented earlier. There was no significant effect of participants’ AoA on RTs to non-signs.

### 3.2. Results on Accuracy

#### 3.2.1. Signs

**Figure 3:**
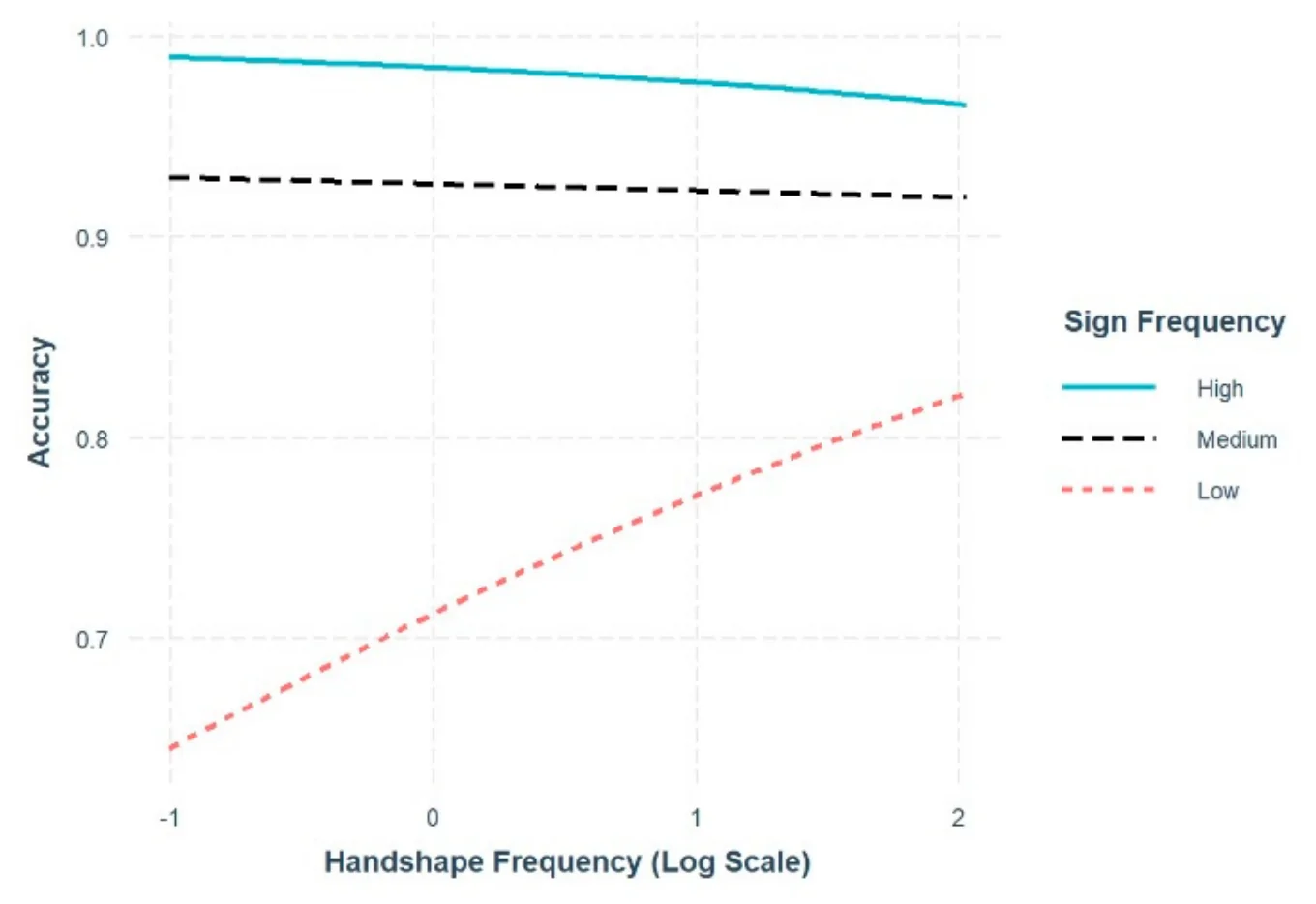
Illustration of the interaction between handshape density and subjective sign frequency in response accuracy to real signs. The continuous variable sign frequency was categorized into low, medium, and high for illustration purposes only.

Models on accuracy of real signs had almost equal fit with analyses using movement density based on combined movement (AIC = 5280.60), path and internal movement as separate predictors (AIC = 5279.83), and path movement only (AIC = 5280.43). The largest difference was ΔAIC = 0.77, well below the conventional threshold of 2, indicating that the three models are essentially indistinguishable in fit. All three models were checked for results, and they all showed a main effect of subjective lexical frequency as well as an interaction between handshape density and subjective lexical frequency. Signs with higher lexical frequency were responded to more accurately than those with lower frequency. The interaction between handshape density and subjective lexical frequency is visualized in Figure 3, showing how mean accuracy varies with handshape density at different levels of sign frequency (low, medium, and high). It indicates that signs with larger handshape density had higher accuracy than signs with smaller handshape density only for less frequent signs. These effects remained significant in all 23 iterations according to the leave-one-subject-out analysis. There were no significant effects of movement density in all three models, suggesting that the null effects on real sign RTs are robust to how it is operationalized. Results of the model with combined movement (marginal R^2^ = 0.123; conditional R^2^ = 0.475) were summarized and presented in Table 4 for illustration and for better comparison with results on RTs.

#### 3.2.2. Non-Signs

Models on accuracy data of the non-signs showed equivalent fit with different treatments of movement (model with combined movement density: AIC = 7183.08; model with path and internal movement density as separate predictors: AIC = 7182.89; model with path movement density only: AIC = 7181.15; maximum ΔAIC = 1.93). Results were checked on all three models, which all showed a significant effect of trial number and no significant effects of other predictors or covariates. They all indicated that participants responded to non-signs more accurately as the experiment progressed. The coefficients of the model with combined movement density (marginal R^2^ = 0.016; conditional R^2^ = 0.394) were summarized in Table 5 for illustration.

### 3.3. Results on the Whole Data: Effects of Lexicality

A different set of mixed-effect models was used to model the effects of lexicality using the entire dataset, including both real signs and non-signs. The linear model on RTs included lexicality (real signs coded as 0.5, non-signs coded as −0.5) as the main predictor, along with stimulus duration, trial number, and participants’ AoA as continuous covariates to control for potential confounding effects of stimulus length, practice/fatigue across the experiment, and participants’ language experience. The logistic model on accuracy used the same fixed effect structure. Both models contained random intercepts for participant and stimulus. The linear model shows substantial random-effect contribution (R^2^m = 0.107, R^2^c = 0.569), while the logistic model yields lower overall explanatory power (R^2^m = 0.037, R^2^c = 0.339) typical of variance-constrained binary outcomes.

In the LME model on RTs, both duration (B = 0.030, β_std = 0.184, SE = 0.002, CI [0.026, 0.033], *t* = 15.950, *p* < 0.001) and trial number (B = −0.027, β_std = −0.166, SE = 0.001, CI [−0.028, −0.025], *t* = −31.525, *p* < 0.001) were significant predictors, indicating slower responses for longer stimuli and a speeding of responses over the course of the experiment. Critically, after controlling for these covariates, the effect of lexicality remained significant (B = −0.043, β_std = −0.132, SE = 0.004, CI [−0.050, −0.035], *t* = −11.477, *p* < 0.001), with real signs responded to faster than non-signs. In the logistic model on accuracy, only lexicality reached significance (OR = 0.786, β_std = 0.393, SE = 0.097, CI [0.597, 0.975], *z* = 8.131, *p* < 0.001), with real signs recognized more accurately than non-signs.

These findings are consistent with those from spoken word recognition (Bien et al., 2015; Dumay et al., 2001; Hunter, 2013; Tucker et al., 2019) and suggest that participants did distinguish between real signs and non-signs in the current task.

## 4. Discussion

The present study examined the influence of neighborhood density defined by three major phonological parameters on CSL lexical recognition as well as the moderating effect of subjective lexical frequency. Results showed that (1) subjective lexical frequency facilitated both RTs and accuracy of responding to real signs; (2) location density inhibited lexical decision times for both signs and non-signs; (3) handshape density facilitated lexical decision accuracy for only real signs with lower frequency; (4) movement density did not produce significant effects on RTs or accuracy of responding to signs or non-signs. These effects replicate previous studies showing that the parameters are used differently in the process of sign recognition (Carreiras et al., 2008; Dye & Shih, 2006; Gutiérrez et al., 2012a) and implicate some cognitive mechanisms shared across language modalities, which will be discussed below in more detail.

### 4.1. Effects of Lexical Frequency

There was a significant main effect of subjective lexical sign frequency on both RTs and accuracy. That lexical items with higher frequency were recognized faster and more accurately than those with lower frequency aligns well with findings from both previous spoken language studies (Brysbaert et al., 2018; Ernestus & Cutler, 2015; Ferrand et al., 2018; Hauk & Pulvermüller, 2004) and sign language studies (Carreiras et al., 2008; Caselli et al., 2021). It demonstrates that language users are sensitive to how frequently a lexical item occurs in a language, whether the language is conveyed in an aural-oral modality or a visual-gestural modality; that is, lexical frequency is psychologically important across language modalities.

Explanations used to account for lexical frequency effects in spoken word recognition models may also be applicable here, including higher resting activation levels (Marslen-Wilson, 1987), stronger phoneme-to-word connections (McClelland et al., 2006; McClelland & Elman, 1986), or a bias of the decision units at the late processing stage (Luce & Pisoni, 1998) for higher frequency lexical items compared to lower frequency items. Although behavioral data alone may not suffice to disentangle these accounts, the cross-modal nature of lexical frequency effects suggests that some general processes may govern lexical recognition in natural human languages irrespective of language modality.

The lexical frequency effect simultaneously supports that the stimulus materials and the lexical decision task used in the present study are effective in probing lexical processing. It serves as a positive test case, demonstrating that participants indeed processed lexical representations rather than relying solely on low-level visual features and providing a logical foundation for interpreting the parameter-specific density effects.

### 4.2. Effect of Location Neighborhood Density

Lexical decision responses were delayed with more location neighbors for both signs and non-signs. This is in line with the inhibitory effects of PND generally reported in spoken word recognition (Dufour & Frauenfelder, 2010; Dufour & Peereman, 2003; Goh et al., 2009, 2016; Luce & Large, 2001; Luce & Pisoni, 1998; Magnuson et al., 2007; Strauß et al., 2022; Vitevitch, 2002; Winsler et al., 2018). The effect patterns are consistent with the multiple activation-competition hypothesis assumed in most spoken word recognition models (Gaskell & Marslen-Wilson, 1997, 2002; Grossberg, 2003; Luce & Pisoni, 1998; McClelland & Elman, 1986; Norris, 1994; Norris & McQueen, 2008). Specifically, as a target sign is presented, its sublexical unit of location is also activated and subsequently activates a set of matching lexical candidates in memory, which compete with the target sign and inhibit its activation rate. Signs sharing location with many other signs may suffer from greater competition and inhibition, leading to response delay in behavior.

The effect also replicates the inhibitory effect of location density reported by Carreiras et al. (2008) and is consistent with interference of location overlap found in prior priming studies (Corina & Hildebrandt, 2004; Gutiérrez et al., 2012a, 2012b). It also aligns well with the strong competitor assumption of Caselli and Cohen-Goldberg’s (2014) computational model. According to this model, location has a longer activation window and a higher resting activation level due to earlier perception upon input (Emmorey & Corina, 1990), earlier acquisition (Bonvillian & Siedlecki, 2000; Conlin et al., 2000; Karnopp, 2002; Marentette & Mayberry, 2000; Morgan et al., 2007; Ortega & Morgan, 2015; Siedlecki & Bonvillian, 1993), higher visual salience (Orfanidou et al., 2009), and higher sublexical frequency in the lexicon (Carreiras et al., 2008; Caselli et al., 2017; Gutierrez-Sigut et al., 2016). This set of inherent advantages may explain why location neighbors exerted substantial competition in the current data, as their chronically elevated baseline activation may make them persistently strong competitors during lexical access, thus prolonging response latencies for target signs sharing this parameter.

However, no interactions were found here between location neighborhood density and subjective lexical frequency, in contrast to Carreiras et al.’s (2008) study, which showed a stronger effect of location on less frequent signs. It is possible that location competition is strong enough to override frequency-based differences in lexical activation, especially considering the extremely high frequency of one location value, i.e., neutral space. This value comprised seven times more neighbors than handshape values in dense neighborhoods in Carreiras et al.’s study, and comprised over 50% of the signs in the current study. It may activate a large number of neighbors and lead to competition robust enough to override lexical frequency modulation.

Then why did location density inhibit responses to non-signs, which have no lexical representations in the mental lexicon and should have no competition to slow down their recognition? Phonological neighbors were defined in this study by shared parameter values. Neighborhood density defined in this way is almost identical to the sublexical frequency of the parameter values, a measure of phonotactic probability, making it difficult to fully separate lexical and sublexical contributions to the location density effect on non-signs. The effect, however, seems unlikely to be based purely on sublexical processing. If the effect were sublexical, high frequency of location values should have facilitated rather than inhibited responses to non-signs, by analogy with the facilitatory effect of phonotactic probability on non-words in spoken languages (Vitevitch, 2003; Vitevitch et al., 1999; Vitevitch & Rodríguez, 2005). Instead, the inhibitory pattern observed here is more consistent with lexical competition. Although non-signs have no lexical representations, their location is compatible with existing CSL signs and would thus activate real sign neighbors. These neighbors may compete with one another at the lexical level, thus increasing uncertainty in the “no” response to non-signs and slowing rejection.

### 4.3. Effect of Handshape Neighborhood Density

Handshape neighborhood density showed a facilitative effect on the accuracy of identifying real signs with lower subjective lexical frequency; that is, accuracy in response to real signs was higher when they had many handshape neighbors. This replicates prior findings of facilitation by handshape density (Ann et al., 2011; Carreiras et al., 2008) and priming (Meade et al., 2021, 2022).

Previous spoken language studies have attributed the facilitatory effects of neighborhood density to an impact of phonotactic probability and argued that these effects result from representation and processing at the sublexical level (Ferrand et al., 2018; Magnuson et al., 2007; Vitevitch, 2003; Vitevitch et al., 1999). However, facilitation would also be found for non-signs if the effect observed here occurred at the sublexical level. In addition, facilitation at the sublexical level is argued to occur early in the process (Dufour et al., 2013; Hunter, 2013), but the facilitative effect of handshape density was found only on response accuracy and not on RTs. In other words, for correct responses, deaf participants’ speed did not vary based on the handshape density of the signs. This suggests that the effect may occur in the later stages, probably reflecting a strategic bias toward signs sharing handshape values with many neighbors at the late decisional stage of the lexical decision task.

This interpretation aligns with predictions within Caselli and Cohen-Goldberg’s (2014) model, where handshape neighbors are assumed to be weak competitors due to later perception and a lower resting activation level of handshape. It implies that when a sign is processed, handshape neighbors do not trigger strong lexical competition. Instead, the visual and kinematic form of the handshape values shared by many signs may feel more typical and familiar to participants. This typicality and familiarity may be used by the decision system as cues for “sign-likeness”, thus improving accuracy of “yes” responses to real signs, especially for the lower-frequency signs with relatively weak lexical activation. Higher-frequency signs have stronger lexical activation and can reach decision threshold more easily without relying on the typicality and familiarity cues, hence no additional effect of handshape density on higher-frequency signs.

The advantage of more typical handshape values may be counteracted by the correct “no” responses toward non-sign targets, leading to null behavioral effects on non-signs. This interpretation reconciles with the seemingly contradictory finding by Gutiérrez et al. (2012a). They observed a larger late negativity (600–800 ms) in the ERP for non-sign targets sharing handshape overlap with primes. The enhanced late negativity may reflect the neural cost of suppressing the misleading cues of more familiar handshape values, which was not captured in the current behavioral measure.

Although the present findings support a late-decisional strategic bias, they do not rule out the possibility that handshape density also influences early processing stages. Future research using more sensitive measures could examine handshape density effects across different stages of lexical recognition.

### 4.4. Lack of Movement Neighborhood Density Effects

Different analyses were carried out in the present study using three distinct treatments of movement, considering controversies in movement representations in linguistic phonological models (Brentari, 1998; Sandler, 1986; van der Kooij, 2002). One combined path and hand internal movement into a single movement category according to the treatment of movement as a unitary phonological parameter in the Prosodic Model (Brentari, 1998), another added path and hand internal movement as two separate and independent categories, and the last one used path movement only (Dye & Shih, 2006). Model comparisons showed better fit for sign RTs with the combined movement measure, whereas the three operationalizations were nearly indistinguishable for sign accuracy, non-sign RTs, and non-sign accuracy. None of these models showed significant effects of movement density on behavioral RTs or accuracy in CSL sign recognition.

The lack of movement density effects may be interpreted within the model proposed by Caselli and Cohen-Goldberg (2014). This model hypothesizes that the influence of phonological parameters on lexical recognition would be determined by their perceptual timing and resting activation levels. Movement has been found to be perceived more slowly than location and handshape (Emmorey & Corina, 1990; Morford & Carlson, 2011), and may thus comprise even weaker competitors than handshape according to the model’s predictions. Then why did movement density not yield facilitation as handshape density did? Movement may be less relied upon by signers in lexical access and recognition, as it is more ambiguous and easier to be misperceived in sign spotting (Orfanidou et al., 2009). It also tends to have more articulatory variability than location and handshape. The same dynamic movement, e.g., straight path movement, may vary in speed, size, and rhythm in different signs and contexts. Classifiers in particular, i.e., polymorphemic signs that are highly variable and weakly lexicalized (Brentari & Padden, 2001), have great variability in movement and may undertake more complex movement features. This articulatory variability may lead to a limited role of movement in isolated lexical recognition investigated in the current study, leading to null effects of movement density here. This echoes previous findings that movement was retrieved less frequently than location and handshape in circumstances of “tip of the fingers”, the signed equivalent of tip of the tongue (R. Thompson et al., 2005), and that when asked to produce as many signs with a given location, handshape, or movement value as possible, signers were less likely to produce sign clusters with movement overlap than clusters with location and handshape overlap (Marshall et al., 2014).

Though these results do not provide evidence for an independent movement effect in this task, they do not rule out a role for movement in other tasks or at other processing levels. Sign language linguists have argued that movement is the most visually sonorous part of a sign and heavily involved in prosodic structures (Brentari, 1998; Johnson & Liddell, 2010; Sandler, 1989), which should be expected to be used during sign language processing. It may contribute more to higher levels of language processing where prosodic and morphological features need to be processed than isolated lexical recognition. It is also plausible that the effect of movement density has not been sufficiently detected in the current experimental paradigm, as one prior priming study did show movement priming effects at an early stage (Dye & Shih, 2006). Further research is needed to more finely probe contributions of movement during the dynamic process of lexical processing, especially with more sensitive measurements like ERPs or eye-tracking techniques.

To summarize, the present study shows distinct processing effects of the three major phonological parameters during CSL sign recognition. However, the findings should be interpreted in light of several limitations, which also point to directions for future research. For one thing, the sample size of deaf signers was relatively small (N = 23) and participants were recruited from a single geographic location, which limits the generalizability of the findings. Future research with larger and more diverse samples is needed. For another, the present study used a frequency measure based on deaf CSL signers’ ratings rather than objective corpus frequency. Subjective frequency ratings may capture not only how often a sign occurs in the language but also how familiar it is to the individual. Consistent with this, the subjective CSL frequency ratings correlated only modestly with spoken Chinese frequency (r = 0.353). This limitation should be borne in mind when interpreting the subjective frequency effects. Future research with corpus-based frequency estimates will help validate the present findings. In addition, the present study employed a single experimental task that cannot capture all aspects of lexical processing. Different tasks (e.g., priming, picture naming, eye-tracking) and techniques (e.g., ERPs) may reveal different patterns of parameter-specific effects. Future research using multiple tasks and techniques will be needed to establish the generality of the present findings.

## 5. Conclusions

This study primarily aims to find out the roles of location, handshape, and movement parameters in CSL sign recognition by examining whether and how their neighborhood density would impact the RTs and accuracy in performing a lexical decision task. Location density showed an inhibitory effect on RTs to both signs and non-signs. Handshape density had a facilitatory effect on response accuracy of real signs. Movement density, however, had no significant impact on the task performance. The results together with previous work indicate that the parameters do play different roles during lexical sign recognition. They are also consistent with core assumptions of Caselli and Cohen-Goldberg’s (2014) model that differences in perceptual timing and resting activation levels influence whether phonological parameters constitute strong or weak competitors during sign recognition and ultimately determine the effect direction of the parameter neighbors. In addition to these parameter-specific effects, subjective lexical frequency robustly facilitated RTs and accuracy in performing the task, replicating the classic lexical frequency effect. The concurrent observation of lexical frequency effects in sign and spoken languages is consistent with the possibility that some principles of lexical processing are shared across modalities, that is, the quantitative properties of language experience (usage frequency) can exert qualitative influences on lexical representations (processing efficiency).

Taken together, these findings point to some principles of lexical processing that may be shared across spoken and sign languages, including the representation and activation of sublexical phonological units, the multiple activation and competition among phonologically similar neighbors, and the modulating role of lexical frequency. However, further research across multiple sign languages, tasks, and larger samples is needed to establish the generality of these mechanisms.

## Figures and Tables

**Table 1:** Characteristics of the real signs and pseudo-signs used in the experiment.

Variable	Mean	Range	SD
Location density			
signs	215.10	2–352	160.90
non-signs	175.55	3–352	164.55
Handshape density			
signs	38.48	1–103	33.65
non-signs	30.28	1–103	30.36
Path movement density			
signs	132.92	2–179	50.08
non-signs	135.72	2–179	48.99
Internal movement density			
signs	387.09	5–508	204.78
non-signs	428.56	5–508	176.86
Combined movement density			
signs	83.07	1–162	59.32
non-signs	96.07	2–162	57.77
Frequency			
signs	5.50	1–7	1.33
non-signs	NA	NA	NA
Iconicity			
signs	4.0128	1.00–7.00	1.88
non-signs	NA	NA	NA
Duration (ms)			
signs	852.26	290–2018	327.79
non-signs	1089.46	363–3024	383.05

Note. Density values represent the number of signs in the CSL lexical database that share the same value for the given parameter. Frequency is the mean subjective frequency rating by 29 native CSL signers (ICC(2, k) = 0.986, 95% CI [0.983, 0.989]) based on a 7-point scale (1 = very rarely, 7 = very frequently). Iconicity is the mean iconicity rating by 31 hearing non-signers based on a 7-point scale (1 = not iconic at all, 7 = very iconic). Duration is the latency between sign onset and offset.

**Table 2:** Summary of LME model results on reaction times of real signs.

Predictors	Reaction Time					
	B	β_std	SE	CI	*t*	*p*
(Intercept)	3.072 ***	NA	0.053	2.968–3.176	58.058	**<0.001**
Location density	0.005 *	0.038	0.003	0.000–0.011	2.032	**0.043**
Handshape density	0.003	0.020	0.003	−0.002–0.008	1.068	0.286
Movement density	−0.004	−0.027	0.003	−0.009–0.001	−1.449	0.148
Frequency	−0.021 ***	−0.141	0.003	−0.028–−0.014	−6.233	**<0.001**
Location density: Frequency	0.001	0.007	0.003	−0.004–0.006	0.370	0.711
Handshape density: Frequency	−0.000	−0.001	0.003	−0.006–0.005	−0.065	0.948
Movement density: Frequency	−0.004	−0.025	0.003	−0.009–0.002	−1.342	0.181
Iconicity	0.005	0.037	0.003	−0.000–0.010	1.947	0.052
Sign Duration	0.026 ***	0.184	0.004	0.018–0.035	6.137	**<0.001**
Trial Number	−0.000 ***	−0.156	0.000	−0.000–−0.000	−19.322	**<0.001**
AoA	−0.007	−0.144	0.006	−0.020–0.006	−1.132	0.269

Note: Frequency = subjective frequency ratings (mean of 29 native signers; ICC(2, k) = 0.986, 95% CI [0.983, 0.989]); AoA = age of acquisition; B = unstandardized regression coefficients; β_std = standardized coefficients; SE = standard error; CI = 95% confidence interval; * *p* < 0.05, *** *p* < 0.001; significant *p* values are shown in bold.

**Table 3:** Summary of LME model results on reaction times of non-signs.

Predictors	Reaction Time					
	B	β_std	SE	CI	*t*	*p*
(Intercept)	3.134 ***	NA	0.059	3.018–3.249	53.142	**<0.001**
Location density	0.006 **	0.043	0.002	0.002–0.010	2.651	**0.008**
Handshape density	−0.001	−0.008	0.003	−0.007–0.004	−0.492	0.623
Movement density	0.002	0.011	0.003	−0.003–0.008	0.725	0.469
Duration	0.025 ***	0.178	0.002	0.021–0.029	11.925	**<0.001**
Trial Number	−0.000 ***	−0.181	0.000	−0.000–−0.000	−20.916	**<0.001**
AoA	−0.005	−0.094	0.007	−0.018–0.009	−0.667	0.511

Note. AoA = age of acquisition; B = unstandardized regression coefficients; β_std = standardized coefficients; SE = standard error, CI = 95% confidence interval; ** *p* < 0.01, *** *p* < 0.001; significant *p* values are shown in bold.

**Table 4:** Summary of logistic mixed-effect model results on accuracy of real signs.

Predictors	Accuracy					
	Odds Ratios	β_std	SE	CI	*z*	*p*
(Intercept)	9.261 ***	NA	5.619	2.820–30.416	3.668	**<0.001**
Location density	1.041	0.040	0.080	0.896–1.210	0.530	0.596
Handshape density	0.946	−0.056	0.071	0.816–1.095	−0.747	0.455
Movement density	0.982	−0.018	0.073	0.850–1.136	−0.240	0.810
Frequency	2.190 ***	0.803	0.176	1.872–2.563	9.770	**<0.001**
Location density: Frequency	1.111	0.108	0.082	0.961–1.285	1.425	0.154
Handshape density: Frequency	0.841 *	−0.178	0.062	0.728–0.971	−2.360	**0.018**
Movement density: Frequency	0.966	−0.036	0.069	0.839–1.111	−0.487	0.627
Iconicity	1.086	0.082	0.082	0.937–1.260	1.094	0.274
Sign Duration	0.909	−0.096	0.067	0.786–1.050	−1.299	0.194
Trial Number	0.908 **	−0.097	0.034	0.844–0.977	−2.599	**0.009**
AoA	1.044	0.124	0.074	0.909–1.199	0.612	0.541

Note. Frequency = subjective frequency ratings (mean of 29 native signers; ICC(2, k) = 0.986, 95% CI [0.983, 0.989]); AoA = age of acquisition; β_std = standardized coefficients; SE = standard error; CI = 95% confidence interval; * *p* < 0.05, ** *p* < 0.01, *** *p* < 0.001; significant *p* values are shown in bold.

**Table 5:** Summary of logistic mixed-effect model results on accuracy of non-signs.

Predictors	Accuracy					
	Odds Ratios	β_std	SE	CI	*z*	*p*
(Intercept)	9.638 ***	NA	6.475	2.583–35.965	3.372	**<0.001**
Location density	1.026	0.026	0.068	0.902–1.168	0.394	0.693
Handshape density	0.998	−0.002	0.082	0.850–1.172	−0.025	0.980
Movement density	1.047	0.033	0.089	0.886–1.237	0.541	0.588
Sign Duration	1.045	0.043	0.065	0.925–1.179	0.706	0.480
Trial Number	1.107 **	0.102	0.034	1.042–1.176	3.286	**0.001**
AoA	0.911	−0.27	0.071	0.781–1.061	−1.198	0.231

Note. AoA = age of acquisition; β_std = standardized coefficients; SE = standard error, CI = 95% confidence interval; ** *p* < 0.01, *** *p* < 0.001; significant *p* values are shown in bold.

## Data Availability

The full CSL lexical database is not publicly available at this stage because it is being prepared for a separate publication. However, the complete stimulus metadata for all stimuli used in the present study are provided in the Supplementary Materials. Further inquiries can be directed to the corresponding author.

## Supplementary Materials

The following supporting information can be downloaded at: https://www.mdpi.com/article/10.3390/bs16101937/s1, Data S1: Metadata for the stimuli used in the current study; Data S2: Dataset used for analyses; Script S1: R scripts for main analyses.

## Abbreviations

The following abbreviations are used in this manuscript:

AIC	Akaike Information Criterion
AoA	Age of acquisition
CSL	Chinese Sign Language
PND	Phonological neighborhood density
RT	Reaction time
LME	Linear mixed-effects
